# Diagnostic accuracy of pleural fluid NT-pro-BNP for pleural effusions of cardiac origin: a systematic review and meta-analysis

**DOI:** 10.1186/1471-2466-10-58

**Published:** 2010-11-20

**Authors:** Surinder Janda, John Swiston

**Affiliations:** 1 Division of Respiratory Medicine, The University of British Columbia, Vancouver, BC, Canada

## Abstract

**Background:**

Several studies have been published in the literature on the diagnostic accuracy of NT-pro-BNP for pleural effusions from heart failure in the last decade. The purpose of our study was to perform a systematic review and meta-analysis on the diagnostic accuracy of pleural fluid NT-pro-BNP for pleural effusions of cardiac origin.

**Methods:**

MEDLINE, EMBASE, PapersFirst, and the Cochrane collaboration and the Cochrane Register of controlled trials were searched. All searches were inclusive as of March 2010. Studies were only included if the absolute number of true-positive, false-negative, true-negative, and false-positive observations were available, and the "reference standards" were described clearly. Two investigators independently reviewed articles and extracted data. Quality was assessed with the Quality Assessment for Diagnostic Accuracy Studies (QUADAS). The bivariate model for diagnostic meta-analysis was used to obtain a pooled sensitivity and a pooled specificity.

**Results:**

Ten studies (total number of patients 1120) were included in the meta-analysis. The average pleural fluid NT-pro-BNP level in effusions of cardiac origin was 6140 pg/mL. The pooled sensitivity and specificity of all studies combined was 94% (95% CI: 90-97) and 94% (95% CI: 89-97) respectively. The pooled positive likelihood ratio was 15.2 (95% CI: 8.1-28.7) and the pooled negative likelihood ratio was 0.06 (95% CI: 0.03-0.11). The area under the ROC curve was 0.98 (95% CI: 0.96-0.99) and the diagnostic odds ratio was 246 (95% CI: 81-745).

**Conclusions:**

Pleural fluid NT-pro-BNP is a very useful biomarker with high diagnostic accuracy for distinguishing pleural effusions of cardiac origin.

## Background

Pleural effusions arising from heart failure are usually discriminated from other causes based on clinical criteria in association with biochemical analysis, particularly the discrimination of transudates versus exudates, most commonly using Light's criteria[1]. The sensitivity of Light's criteria for identifying exudative pleural effusions is very high (98%)[2], however the criteria's ability to exclude transudative effusions is low[3]. As a result, heart failure associated pleural effusions can be misclassified as exudates using Light's criteria, particularly after diuretics have been used. One study showed that 28% (5 of 18) patients with pleural effusions from congestive heart failure (CHF) were misclassified as exudative effusions using Light's criteria[4]. A diagnostic dilemma often arises when CHF-associated pleural effusions are misclassified as exudates which can then lead to the use of more expensive and sometimes invasive tests to diagnose the etiology of the effusion. A non-invasive and inexpensive strategy to discriminate pleural effusions of a cardiac origin would be beneficial in such circumstances.

Serum brain natriuretic peptide (BNP) or the amino-terminal fragment N-terminal pro-brain natriuretic peptide (NT-pro-BNP) have an established role in the diagnosis, management, and prognosis of patients with CHF[5]. BNP, also known as B-type natriuretic peptide, is a 32 amino acid polypeptide secreted by the ventricles of the heart in response to excessive stretching of cardiomyocytes[6]. BNP is co-secreted along with a 76 amino acid polypeptide, NT-pro-BNP, which is biologically inactive[7]. The half-life of BNP is approximately 20 minutes whereas of NT-pro-BNP is 1-2 hours[8]. BNP binds to atrial natriuretic factor receptors leading to a decrease in systemic vascular resistance and central venous pressure, and an increase in natriuresis[6].

Several studies have been published in the literature on the diagnostic accuracy of NT-pro-BNP for pleural effusions from heart failure. The purpose of our study was to perform a systematic review and meta-analysis on the diagnostic accuracy of pleural fluid NT-pro-BNP for pleural effusions of cardiac origin.

## Methods

The systematic review and meta-analysis was performed according to the recently published recommendations and checklist of the PRISMA statement[9].

Searches were conducted on MEDLINE (inception-March 2010); EMBASE (inception-March 2010), PapersFirst (inception-March 2010), and the Cochrane collaboration and the Cochrane Register of controlled trials for relevant studies. The following key terms were used: 'pleural fluid' or 'pleural effusion' AND 'brain natriuretic peptide' or 'B-type natriuretic peptide' or 'BNP', 'pro-BNP' or 'NT-pro-BNP' or 'amino-terminal pro-BNP' or 'congestive heart failure' or 'heart failure' or 'CHF'. All searches were limited to 'humans'. We identified additional studies by searching the bibliographies of retrieved articles. Two independent reviewers (SJ and JS) performed the literature search. Studies relevant to the diagnostic value of NT-pro-BNP for pleural effusions of cardiac origin were included if the following criteria were met: pleural fluid NT-pro-BNP was used for diagnosing pleural effusions of cardiac origin; and a 2 × 2 contingency table could be formulated from the available data. Studies that used BNP or other biomarkers were excluded.

All studies that appeared to fit the inclusion criteria were identified for full review by two reviewers (SJ and JS). Each reviewer independently selected studies for inclusion in the review. Disagreement between the two extracting authors was resolved by consensus.

The methodological quality of the selected studies was graded independently by two reviewers (SJ and JS) with the Quality Assessment of Diagnostic Accuracy Studies (QUADAS) tool, a validated tool for the quality assessment of diagnostic accuracy studies[10]. We performed component analysis using the QUADAS tool which was depicted as a proportional bar graph for each of the 14 individual criteria. Disagreement between the two extracting authors was resolved by consensus.

The following variables were extracted from each study: publication year; country of origin of study; study design; patient demographics and co-morbidities; NT-pro-BNP assay type; numbers of true-positive, false-negative, true-negative, and false-positive observations; correlation statistic between pleural fluid and serum NT-pro-BNP if available; and the reference standard used.

We used the bivariate model for diagnostic meta-analysis to obtain an overall sensitivity and an overall specificity[11]. Instead of using the diagnostic odds ratio, as used in conventional diagnostic meta-analysis[12], the bivariate model uses pairs of sensitivity and specificity as the starting point of the analysis. In addition to accounting for study size, the bivariate model estimates and incorporates the negative correlation that may arise between the sensitivity and specificity of the index test within studies as a result of differences in test threshold between studies. The bivariate model uses a random effects approach for both sensitivity and specificity, which allows for heterogeneity beyond chance as a result of clinical and methodological differences between studies. The pooled estimates of sensitivity and specificity were used to calculate the average positive and negative likelihood ratios. Publication bias through small study effects was assessed with a regression test on the diagnostic odds ratio[13].

A receiver operating characteristic graph, with the y-axis representing the index test's sensitivity (true positive rate) and the x-axis representing 1-specificity (false negative rate), was used to plot the individual and summary points of sensitivity and specificity. Furthermore, around the pooled estimates, we also plotted a 95% confidence region and a 95% prediction region to illustrate the precision with which the pooled values were estimated (confidence ellipse of a mean) and to show the amount of between study variation (prediction ellipse; the likely range of values for a new study). We used Stata intercooled version 10.1 (StataCorp, College Station, Texas), in particular the *midas *and *metandi *commands, for all statistical analyses[14,15].

## Results

**Figure 1:**
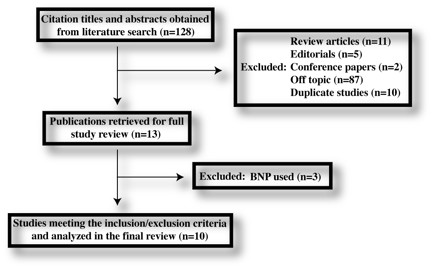
**Flow chart of the studies included in the meta-analysis**.

Our search yielded 128 citations of which 117 were excluded for various reasons based on the title and abstract (Figure 1). Eleven were then retrieved for full text review of which three were excluded because BNP instead of NT-pro-BNP was used as the biomarker (Figure 1). Ten studies were included in the final analysis[16-25]. One study, by Seyhan *et al*[22], was initially published online and then was retracted by the editors of the journal. The reason for retraction was due to a violation of the journal's *Information for Authors*. Therefore, only the abstract was available for data extraction and a quality assessment was not possible.

The studies were published from 2004 to 2010. The majority of the studies (7/9)[16-19,22-24] were done in Europe whereas two were done in North America[20,25] and one in Asia[21]. All studies except for two (Porcel *et el*[23] and Long *et al*[25]) were of prospective design (Table 1). Furthermore, all studies except for two (Liao *et al*[20] and Long *et al*[25]) used an electrochemical luminescence immunoassay (ELCIA) performed on the Elecsys 2010 analyzer (Roche) to measure levels of NT-pro-BNP whereas Liao *et al*[20] and Long *et al*[25] used an enzyme-linked immunosorbent assay (ELISA) (Table 1). A total of 1120 patients/pleural effusions were included in this analysis (429 of cardiac origin and 691 of non-cardiac origin). The control group (non-cardiac pleural effusions) consisted of mainly malignant pleural effusions and infections (Table 1).

**Table 1:** Characteristics of the studies included in the meta-analysis.

Study	Year	Country	Design	Assay Type	Reference Standard	Control Group^†^
Tomcsanyi *et al*[16]	2004	Hungary	PC	ECLIA(1)^‡^	Clinical Criteria* a, b, c, e	57% M; 14% I; 29% O
Porcel *et al*[17]	2004	Spain	PC	ECLIA(1)	Clinical Criteria* a, b, c, d, e, f	NR
Kolditz *et al*[18]	2006	Germany	PC	ECLIA(1)	Clinical Criteria* a, b, c, e, f	59% M; 21% I; 30% O
Porcel *et al*[19]	2007	Spain	PC	ECLIA(1)	Clinical Criteria* a, b, c, d, e, f	48% M; 20% I; 32% O
Liao *et al*[20]	2008	USA	PC	ELISA	Clinical Criteria* a, c, e	25% M; 0% I; 75% O
Han *et al*[21]	2008	Korea	PC	ECLIA(1)	Clinical Criteria* a, b, e, f	24% M; 66% I; 10% O
Seyhan *et al*[22]	2009	Turkey	PC	ECLIA	Clinical Criteria* N/A	N/A
Porcel *et al*[23]	2009	Spain	RC	ECLIA(2)	Clinical Criteria* a, b, c, e, f	44% M; 29% I; 27% O
Bayram *et al*[24]	2009	Turkey	PC	ECLIA(1)	Clinical Criteria* a, b, c, e, f	23% M; 31% I; 46% O
Long *et al*[25]	2010	USA	RC	ELISA	Clinical Criteria* a, b, c, e	25% M; 25% I; 25% O

* = heart failure diagnosis based on (a) history, or (b) physical exam, or (c) chest x-ray, or (d) electrocardiogram, or (e) echocardiogram, or (f) response to diuretics; † = non-cardiac effusions; ‡ = generation of assay (one or two); ECLIA = electrochemical luminescence immunoassay; ELISA = enzyme-linked immunosorbent assay; I = infectious effusion; M = malignant effusion; N/A = not available; NR = not recorded; O = other causes (i.e. connective tissue disease, pleuritis, post-CABG, hepatic hydrothorax, pulmonary embolism, etc); PC = prospective cohort; RC = retrospective cohort;

As there is no *gold standard *for effusions of cardiac origin, clinical criteria was used as the reference standard. All studies used some combination of the following criteria: history, physical exam, chest x-ray, electrocardiogram, echocardiogram, and response to diuretics. Of the studies[18-20,23,25] that reported New York Heart Classification for the patients with effusions of cardiac origin, all patients were either class III or IV.

**Figure 2:**
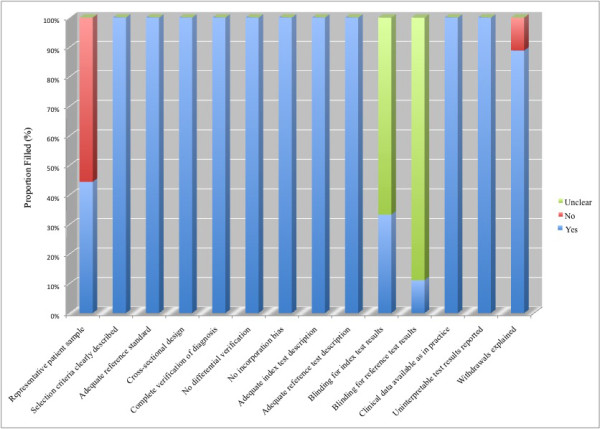
**Proportion of all 14 Quality Assessment of Diagnostic Accuracy Studies (QUADAS) tool criteria that were fulfilled for seven of eight studies included in the meta-analysis**.

Overall, the quality of the reported studies was good (Figure 2). Five studies[16,19,20,23,25] maybe subject to spectrum bias as their method of recruitment of patients consisted of recruiting a target group (patients with pleural effusions of cardiac origin) and a control group (patients with pleural effusions of non-cardiac origin) rather than applying the index and reference test to an unselected patient population with pleural effusions. Most studies may be subject to review bias as it was unclear in eight studies whether the investigators who used the reference test (clinical criteria) were blinded to the results of the NT-pro-BNP assay and six studies[16,18,20,21,24,25] did not state whether the laboratory personnel performing the index test (NT-pro-BNP assay) were blinded to the clinical diagnosis.

The average NT-pro-BNP level for nine of the ten studies was 6140 pg/mL in pleural effusions of cardiac origin (this value was not available for one study[22]). Of the five studies[16,18,19,21,24] that analyzed both serum and pleural fluid NT-pro-BNP levels, all five studies showed high correlation between these two parameters (Table 2). Table 2 also shows that the percentage of cardiac effusions misclassified as exudates by Light's criteria was relatively modest (range 7%-36%) within various studies. However, when these misclassified effusions from Light's criteria were analyzed by NT-pro-BNP levels, the absolute number of misclassified effusions was significantly reduced (Table 2).

**Table 2:** Diagnostic accuracy variables of the studies included in the meta-analysis.

Study	**No**. Pts/Pes	TP	FN	TN	FP	Se	Sp	NT-pro-BNP Threshold*	Average **NT-pro-BNP**^†^	Misclassification		Correlation Statistic^∏^
										Light's^‡^	**NT-pro-BNP**^§^	
Tomcsanyi *et al*[16]	28	14	0	14	0	100%	100%	≥ 599^¶^	8236	1/14 (7%)	0/1	*R^2 ^*= 0.95 p < 0.0001
Porcel *et al*[17]	117	40	4	68	5	91%	93%	≥ 1500	6931	10/35 (29%)	2/10	NR
Kolditz *et al*[18]	93	23	2	63	5	92%	93%	≥ 4000	10427	9/25 (36%)	0/9	*S *= 0.96 p < 0.001
Porcel *et al*[19]	93	49	4	35	5	92%	87%	≥ 1500	6106	8/53 (15%)	2/8	*S *= 0.96 p < 0.001
Liao *et al*[20]	40	10	0	29	1	100%	97%	≥ 2220	5390	NR	NR	NR
Han *et al*[21]	240	81	1	156	2	99%	99%	≥ 1714	3310	28/82 (34%)	1/28	*R^2 ^*= 0.93 p < 0.001
Seyhan *et al*[22]	115	47	4	61	3	92%	95%	≥ 1092	N/A	17/51 (33%)	N/A	N/A
Porcel *et al*[23]	181	84	6	81	10	93%	89%	≥ 1500	6203	20/90 (22%)	4/20	NR
Bayram *et al*[24]	133	48	3	78	4	94%	95%	≥ 925	4468	19/51 (37%)	0/19	*R^2 ^*= 0.91 p < 0.001
Long *et al*[25]	80	16	4	44	16	80%	73%	≥ 2000	4189	NR	NR	NR

* = pleural fluid NT-pro-BNP threshold in pg/mL; † = average pleural fluid NT-pro-BNP in CHF effusions in pg/mL; **‡ **= misclassification of CHF effusions as exudates by Light's criteria; § = misclassification of the CHF effusions already misclassified by Light's criteria now misclassified by NT-pro-BNP; **∏ **= correlation between serum and pleural fluid NT-pro-BNP levels; ¶ = threshold from 599 to 1457 pg/mL resulted in 100% sensitivity and specificity; N/A = not available because only abstract available for data extraction; No. = number; NR = not recorded; Pes = pleural effusions; Pts = patients; *R^2 ^*= Pearson coefficient of correlation; S = Spearman's coefficient of rank correlation; Se = sensitivity; Sp = specificity.

**Figure 3:**
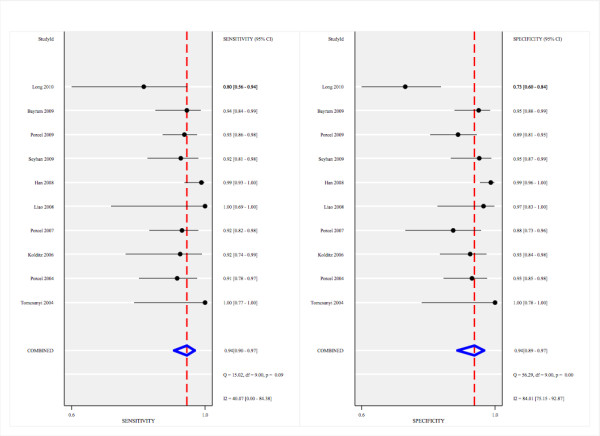
**Forrest plot of the sensitivity and specificity of each individual study, pooled sensitivity and specificity, and *I^2 ^*statistic for heterogeneity**.

**Figure 4:**
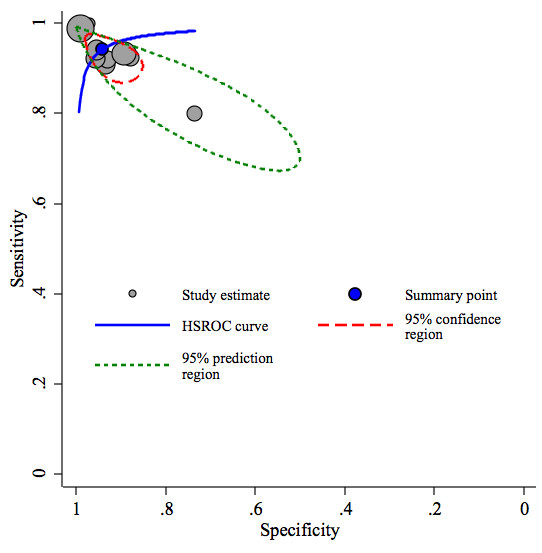
**Summary receiver operating characteristic graph with 95% confidence region and 95% prediction region for NT-pro-BNP**.

The pooled sensitivity and specificity of all studies combined was 94% (95% CI: 90-97) and 94% (95% CI: 89-97) respectively (Figure 3). The pooled positive likelihood ratio was 15.2 (95% CI: 8.1-28.7) and the pooled negative likelihood ratio was 0.06 (95% CI: 0.03-0.11). The area under the ROC curve was 0.98 (95% CI: 0.96-0.99) and the diagnostic odds ratio was 246 (95% CI: 81-745). Figure 4 shows the summary receiver operating characteristic graph with 95% confidence region and 95% prediction region for NT-pro-BNP.

**Figure 5:**
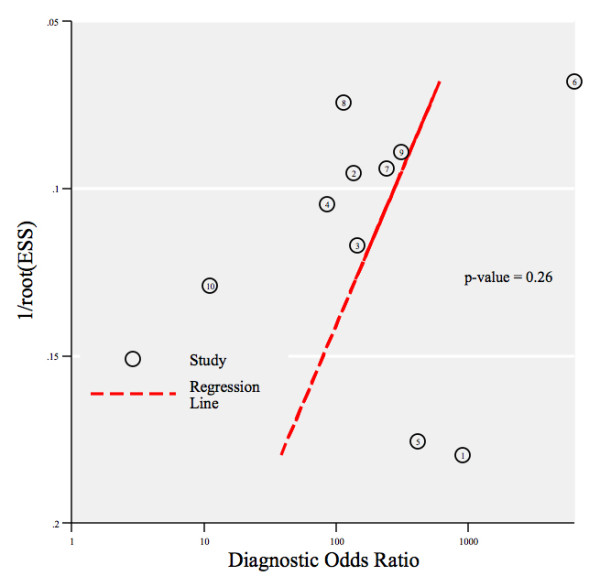
**Deeks' funnel plot asymmetry test for publication bias**.

**Figure 6:**
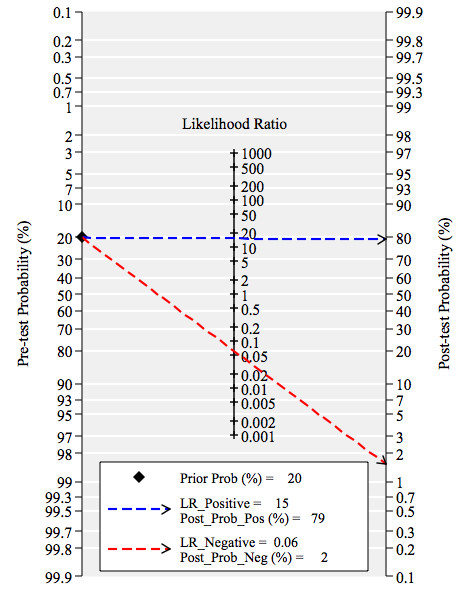
**Fagan's nomogram for NT-pro-BNP illustrating post-test probability with a fixed pre-test probability of 20% for a pleural effusion of cardiac origin**.

The between study variability (i.e. heterogeneity) beyond what could be expected by sampling error was moderate with an *I^2 ^*of 40% (p = 0.09) for the sensitivity results and high with an *I^2 ^*of 84% (p < 0.01) for the specificity results. The bivariate model analysis reveals that this heterogeneity is completely (100%) explained by threshold effect. Analysis of small study effects, potentially a result of publication bias, yielded no significant evidence for such effects with a p value of 0.26 and a funnel plot that was fairly symmetrical (Figure 5). Figure 6 illustrates the post-test probabilities based on various pre-test probabilities using a Fagan nomogram.

## Discussion and Conclusion

Pleural effusions are relatively common in medical practice. It is estimated that the annual incidence of pleural effusions in the United States (US) is 1.5 million cases[26]. The most common cause of pleural effusions is CHF with an estimated incidence of 500,000 cases in the US per year[2]. Identifying the underlying etiology of pleural effusions requires a combination of strategies including clinical history, pleural fluid analysis, and potentially more invasive procedures such as pleural tissue biopsy. Clinical history is very important in the diagnosis of pleural effusions from a cardiac origin. However, alone, it does not appear to be very accurate. Romero-Candeira *et al*[27] studied 64 patients with transudative pleural effusions of which 44 were due to CHF and showed that in 40% of cases initial clinical history failed to correctly classify the effusion. Pleural fluid analysis using Light's criteria is better than clinical history for diagnosing pleural effusions of cardiac origin however these criteria misclassify transudates as exudative effusions approximately 25% of the time, mainly related to the use of diuretics[2]. Misclassification of transudates to exudates can lead to inappropriate management or potentially more invasive diagnostic investigations resulting in increased morbidity and health care costs. As a result, tests with higher diagnostic accuracy may prove beneficial in terms of reducing morbidity and improving cost-efficiency for diagnosing pleural effusions of cardiac origin.

In the last decade several studies have been reported assessing the diagnostic utility of pleural fluid NT-pro-BNP in pleural effusions of cardiac origin. To summarize these studies, we conducted a systematic review and meta-analysis. We included ten studies, of which seven were from Europe, with a total of 1120 patients and/or pleural effusions. We showed that the pooled sensitivity and specificity were identical (94% [95% CI: 90-97] and 94% [95% CI: 89-97]) with a diagnostic odds ratio of 246 (95% CI: 81-745). When the study by Seyhan *et al*[22], which was retracted from the literature, was excluded from the analysis, the sensitivity, specificity, and diagnostic odds ratio remained the same within 95% confidence intervals.

The quality of the studies was generally good overall. The main limitations of the studies were the possibility of spectrum bias, review bias, and population bias. Spectrum bias refers to distortions in a diagnostic test's performance caused by a distortion in the study population. Testing is not done across a population with the expected distribution of disease severity, but rather limited subsets[28]. In our meta-analysis, five studies[16,19,20,23,25] acquired their study population by selecting CHF patients and patients with pleural effusions of non-cardiac origin (control group). The presence of spectrum bias can lead to an overestimation of the sensitivity and specificity of the test[28]. Review bias refers to a situation where persons interpreting the index test have knowledge of the reference standard or vice versa, when persons interpreting the reference standard have knowledge of the index test[29]. In our meta-analysis, it was very unclear whether this did or did not occur because the majority of the studies did not report whether blinding during testing was done. Again, this may have lead to an overestimation of the diagnostic performance of the test. Finally, population bias refers to the generalizability of the diagnostic test to a wider population[30]. The studies included in our meta-analysis had poor reporting of any co-morbid conditions within the CHF-associated pleural effusion group thus potentially limiting generalizability. Furthermore, a thoracentesis in patients with a high pre-test probability for a cardiac origin of the pleural effusion with low probability for other causes would not usually be indicated in most clinical scenarios thus further limiting the generalizability of these results.

Heterogeneity was moderate for the sensitivity results and high for the specificity results (Figure 3). In the summary receiver operating characteristic graph (Figure 4), all but three of the ten 'sensitivity - (1-specificity)' combinations of the individual studies lie on or near the summary receiver operating characteristic curve. This indicates that studies with a higher sensitivity have lower specificity and vice versa. This pattern is commonly attributed to differences in the threshold level for test positivity used in different studies[12]. As a result, the heterogeneity between the studies could well be explained by threshold level differences between the studies which ranged from 599 to 4000 pg/mL. Another potential cause for the heterogeneity may be due to the various types of clinical criteria used for the reference standard (Table 1) as there is no gold standard for the diagnosis of pleural effusions from a cardiac origin. The most objective criteria for heart failure is echocardiography and all studies except for Seyhan *et al*[22] used this parameter and of those studies, the majority defined CHF as an ejection fraction ≤ 40%. Furthermore, of the studies that reported New York Heart Class, the CHF-associated pleural effusion patients were of New York Heart Class (NYHC) III or IV. The control group among the various studies differed and potentially could have contributed to the heterogeneity of the results as well. Malignant pleural effusions and parapneumonic effusions were the main groups that comprised the control groups but some studies did have other causes as a majority of the control group such as connective tissue disease, pleuritis, post-CABG, hepatic hydrothorax, and pulmonary embolism. Finally, eight of the ten studies used electrochemical luminescence immunoassay (ECLIA) for measuring NT-pro-BNP (8 studies generation one, one study generation two, one study unknown generation) while two studies used ELISA which may have added to the heterogeneity of results as well. ECLIA uses a non-competitive immunoassay that produces luminescence via an electrochemical reaction and compared to ELISA, is faster and more precise[31]. The ELISA kit from Biomedica has been compared to the ECLIA kit from Roche Diagnostics revealing significant disparity of results between the two types of assays[31]. The Roche Diagnostics assay (ECLIA) has dual antibodies targeting two different areas of the NT-pro-BNP molecule whereas the Biomedica assay (ELISA) only has a single antibody targeting one region. This may result in better recognition of NT-pro-BNP and hence better diagnostic accuracy for the Roche Diagnostics assay. Furthermore, the cut-off values for serum NT-pro-BNP are 10 fold higher using the Biomedica assay (ELISA) compared to the Roche Diagnostics assay (ECLIA)[31] which may have also added to the heterogeneity of the results.

The correlation between pleural fluid and serum levels of NT-pro-BNP are high as shown by five studies[16,18,19,21,24] in our meta-analysis (Table 2). This suggests that thoracentesis could potentially be avoided in patients with a clinical suspicion of CHF, elevated NT-pro-BNP, and no suspicion for a co-existing cause of the pleural effusion.

Most institutions use BNP to diagnose CHF. Three studies[23,25,32] have examined the diagnostic accuracy of BNP for pleural effusions of cardiac origin. The first study[32] of 57 patients showed a sensitivity of 97% and a specificity of 89% for plasma BNP. The second study by Porcel *et al*[23] (181 patients), showed a sensitivity of 74% and specificity of 92% for pleural fluid BNP and also showed that the area under the curve (AUC) for NT-pro-BNP was higher than for pleural fluid BNP, 0.96 (95% CI: 0.94-0.99) vs 0.90 (95% CI: 0.86-0.95) respectively. Correlation between pleural fluid NT-pro-BNP levels and BNP levels was good, r = 0.78 (p < 0.001). The conclusion from this study was to use NT-pro-BNP rather than BNP for diagnosing pleural effusions of cardiac origin because of the diagnostic superiority of NT-pro-BNP versus BNP, greater in-vitro stability of NT-pro-BNP compared with BNP, and longer half-life of NT-pro-BNP (1-2 hours) compared with BNP (20 minutes). The last study by Long *et al*[25] (80 patients) showed that the AUC for NT-pro-BNP was greater than for pleural fluid BNP, 0.84 (95% CI: 0.72-0.95) vs 0.70 (95% CI: 0.57-0.83). Correlation between pleural fluid NT-pro-BNP and BNP levels was also good but less impressive, r = 0.57 (p < 0.001), and only explained 32% of the variance in NT-pro-BNP. The conclusion from this study was that NT-pro-BNP was a more stable molecule after sample processing and could be maintained for a greater duration in an in vitro setting compared to BNP and furthermore because of the higher diagnostic accuracy of NT-pro-BNP, NT-pro-BNP should be used over BNP to distinguish pleural effusions of cardiac origin in select patient populations.

It is difficult to suggest a threshold level for pleural fluid NT-pro-BNP since the studies used varying threshold levels. The average pleural fluid NT-pro-BNP level in the studies was 6140 pg/mL. Six of the studies[16,17,19,21,23,24] used a threshold level between 1457 to 1714 pg/mL (sensitivity and specificity for a threshold level of 1457 pg/mL by ROC analysis in the study by Bayram *et al*[24] was 84% and 98%, respectively). The pooled sensitivity and specificity of these six studies is 95% (95% CI: 91-98) and 95% (95% CI: 89-98), respectively, with a diagnostic odds ratio of 370 (95% CI: 101-1351). As a result, a threshold level of approximately ≥ 1500 pg/mL provides very good diagnostic accuracy for pleural fluid NT-pro-BNP.

The utility of NT-pro-BNP for the diagnosis of pleural effusions of cardiac origin can be applied to several clinical situations. First, if the pre-test probability of a pleural effusion due to heart failure is high and alternative diagnoses are less likely, then a thoracentesis can be avoided and an elevated serum NT-pro-BNP level will be diagnostic for a cardiac origin of the pleural effusion. Second, if the pre-test probability of a pleural effusion due to heart failure is low or alternative diagnoses are considered in addition to heart failure, then a thoracentesis should be performed and if the pleural fluid NT-pro-BNP level is high then this would be diagnostic of heart failure as one potential cause for the pleural effusion. Furthermore, in undiagnosed pleural effusions, for example when Light's criteria misclassify transudative CHF effusions as exudates and the pre-test probability is equivocal for CHF, a high pleural fluid NT-pro-BNP would also be diagnostic.

The results of our meta-analysis are corroborated by another meta-analysis done by Zhou *et al*[33]. They included eight studies and found that the sensitivity and specificity for NT-pro-BNP for pleural effusions of cardiac origin was 95% (95% CI: 92-97) and 94% (95% CI: 92-96), respectively, with a diagnostic odds ratio of 214 (95% CI: 123-374). Based on their meta-analysis, Zhou *et al*[33] concluded that pleural fluid NT-pro-BNP has high diagnostic accuracy for differentiating cardiac from non-cardiac conditions in patients presenting with pleural effusions.

In summary, misclassification of pleural effusions of cardiac origin can lead to increased morbidity from further invasive testing and increased health care costs. Clinical history and pleural fluid analysis with Light's criteria may not have sufficient diagnostic accuracy to diagnose these effusions in certain clinical circumstances. Although there is some heterogeneity within this analysis, we conclude that where the diagnosis of pleural effusion of cardiac origin is under contemplation, pleural fluid NT-pro-BNP is a useful biomarker with high diagnostic accuracy.

## Abbreviations

BNP: brain natriuretic peptide; CHF: congestive heart failure; ELCIA: electrochemical luminescence immunoassay; ELISA: enzyme-linked immunosorbent assay; HSROC: hierarchical summary receiver operating characteristic; NT-pro-BNP: N-terminal pro-brain natriuretic peptide; QUADAS: quality assessment of diagnostic accuracy studies; US: United States

## Competing interests

Dr. Janda and Dr. Swiston have no competing interests.

## Pre-publication history

The pre-publication history for this paper can be accessed here:

http://www.biomedcentral.com/1471-2466/10/58/prepub
